# Thriving Under Threat: A Scoping Review of Human Thriving in Recurring Potentially Traumatic, Elevated Threat and High Stress Work Environments

**DOI:** 10.1002/smi.70084

**Published:** 2025-07-23

**Authors:** Sally Edmondson, Kemi Wright, Ben Jackson, Aaron Simpson, Bonnie Furzer

**Affiliations:** ^1^ School of Human Sciences (Exercise and Sport Science) The University of Western Australia Crawley Australia; ^2^ School of Health Sciences The University of New South Wales Sydney Australia; ^3^ The Kids Research Institute Australia Perth Australia; ^4^ Fremantle Hospital Mental Health Service South Metropolitan Health Service Fremantle Australia

**Keywords:** coping, flow, law enforcement officers, military personnel, police officers

## Abstract

In this scoping review, we explore the concept of human thriving in work populations that are repeatedly exposed to high stress, elevated threat, and potential trauma—professions such as first responders and front‐line military personnel. The concept of thriving, defined as the joint experience of development and success, shares some similarities with other psychological concepts (e.g., resilience, posttraumatic growth, flourishing), but is distinct due to the consideration of physical wellbeing, and success (e.g., performance). It is posited that thriving offers a more holistic approach to understanding human functioning and is flexible enough to be applied to a variety of populations. We aimed to synthesis the existing literature on human thriving in high stress and recurring trauma occupations, and report factors that enable individuals to thrive. Database searches were conducted in CINAHL, Embase, Medline, and PsycINFO. The review adhered to recommended guidelines including the PRISMA extension for scoping reviews. Eight hundred and thirty‐two original sources were identified and underwent title and abstract review, with 149 retained for full text review and 28 retained for data extraction. Whilst no articles were found that utilised ‘thriving’ as the central concept of investigation in relevant occupational settings (i.e., high stress, recurring trauma), the final sample retained 28 articles that focused on one or more components of thriving (26 quantitative, one mixed method and one qualitative study). Occupations included police officers, paramedics, firefighters, and military personnel. Personal factors identified that align with the thriving framework included resilience, posttraumatic growth and subjective wellbeing. Coping styles also appeared to be related to an individual's ability to thrive with findings suggesting that an active coping style is linked to greater wellbeing, and that an avoidant coping may be helpful during a stressful event. Contextual factors identified included social support from colleagues and supervisors, shared humour, and positive human connection. For individuals in occupations where they are regularly exposed to recurring trauma and stress, and the corresponding negative impacts, finding ways to facilitate thriving may have significant social, psychological, and organisational benefits. Understanding how individuals thrive and positively adapt to disruptions may inform workplace education and interventions and assist in supporting these vital workforces.

## 1 Introduction

Individuals in high stress, high trauma exposure occupations (e.g., first responders, military personnel and emergency doctors) are at a heightened risk of experiencing adverse mental and physical health (Jones 2017; Walker et al. 2016). For instance, individuals in these occupations experience increased rates of depression, anxiety, and posttraumatic stress disorder (Terte and Stephens 2014). Furthermore, these occupations are also characterised by high stakes, high accountability, and involvement in potentially life‐threatening situations. Research attention has often been directed at how posttraumatic stress disorder —and to a lesser extent, resilience—may shape individuals' reactions to stress and trauma in these populations (Brassington and Lomas 2020; Obuobi‐Donkor et al. 2022; Terte and Stephens 2014). However, there remains a relatively limited understanding of how and when these individuals may actually *thrive* in such challenging work environments. Broadly, the concept of thriving refers to an optimal state of human functioning, with a forward or positive momentum. Whilst it shares some similarities with other psychological concepts such as resilience, posttraumatic growth and flourishing, it is distinct due to a number of factors including the inclusion of physical wellbeing, and success (e.g., performance). As a relatively newly articulated concept, there is some variations in the conceptual definitions of thriving, however it presents a valuable concept that is flexible enough to be applied to a variety of populations and offers a more holistic approach to understanding human functioning.

### 1.1 What Is Thriving?

Definitions of thriving often vary by context (see Brown et al. 2017; Goh et al. 2021; Kleine et al. 2019; Spreitzer et al. 2012), with two primary perspectives most commonly described in the literature. Spreitzer and colleagues have proposed a definition and model of thriving stemming from research on work populations (Spreitzer et al. 2012), whilst Brown and colleagues' perspective on thriving has emerged largely from sports‐based research (Brown et al. 2017, 2018, 2021a, 2021b). In work populations, thriving is typically described as ‘the psychological state in which individuals experience both a sense of vitality and a sense of learning at work’ (Spreitzer et al. 2005, 538). These authors suggested that these two key components (i.e., vitality and learning) must function together, and that high levels of both components are necessary for an individual to be deemed to be ‘thriving’ (Goh et al. 2021). Goh et al. (2021) suggest that thriving at work is distinguished from other similar concepts such as job satisfaction because it ‘captures a sense of forward momentum’ (p.1).

Brown and colleagues (2017) defined thriving as ‘the joint experience of development and success’ (p.168), often colloquially considered to reflect ‘living to one's full potential’. These authors suggested the ‘development’ component of thriving is met when an individual experiences high levels of wellbeing (social, psychological and physical) and perceived high performance. When this occurs concurrently with the experience of ‘success’—which is usually outcome‐based, such as school grades or objective work performance indicators—a state of thriving is achieved. If an individual can sustain high wellbeing and performance over a series of situations and over time, this may result in sustained thriving (Brown et al. 2017). These researchers suggested that this definition is flexible enough to include both global (i.e., occurring across pursuits and contexts) and domain‐specific (e.g., within sport or work) conceptualisations, however in order to understand its applicability and utility further research is needed beyond sport and high‐performance occupational settings.

The conceptual framework of thriving defined by Brown and colleagues offers a distinct and potentially valuable approach to provide a holistic understanding of human functioning given it unique focus on success (e.g. high performance) and physical wellbeing which is often overlooked in related concepts (e.g., resilience, flourishing). According to Brown et al. (2017), this physical component of thriving aligns with the notion that development and success are connected to health and physical function. Furthermore, Epel et al. (1998) posit a physiological mechanism that serves as a marker for thriving may exist, theorising that individuals who display cortisol adaptation in the face of repeated trauma exposure may reflect a more robust psychological adaptation. To explore this theory, Brown et al. (2021a, 2021b) investigated cognitive appraisal and hormonal profiles within a sports performance setting and suggested (albeit without statistically significant findings) that the suppression of an individual's acute cortical response may be a manifestation associated with thriving.

### 1.2 How Does Thriving Differ From Related Concepts (e.g., Resilience and Posttraumatic Growth)?

To better understand what thriving is, it is important to also consider what it is not. Terms such as wellbeing, resilience, flow, flourishing, and posttraumatic growth are often used interchangeably and may contribute to ambiguity regarding conceptual differentiation (Brown et al. 2017). Thriving is often considered distinct from resilience in that resilience presupposes exposure to some form of adversity (Brown et al. 2021a, 2021b; Fletcher and Sarkar 2012). Resilience is generally defined as being able to withstand or tolerate adverse, stressful, or traumatic events, and is evidenced through a return to one's ‘normal’ level of function (Connor and Davidson 2003; Fletcher and Sarkar 2012; Southwick et al. 2014). Conversely, thriving is proposed to be possible in response to, or in the absence of, a specific adverse experience, and is postulated as improving or functioning at one's highest level (rather than returning to one's previous level of functioning). In that sense, Brown et al. (2017) emphasised that a high degree of resilience following adversity may act as a personal factor (i.e., determinant) that *contributes* to thriving. Flow is a state in which an individual is completely immersed in the task at hand to the exclusion of any distractions (Stamatelopoulou et al. 2018). A flow state involves immediate feedback, a sense of control, and the experience that being involved in the activity is a reward in itself (Norsworthy et al. 2021). Spreitzer et al. (2005) delineate flow from thriving, arguing that ‘people can be in a flow state and not see themselves as learning’ (2005, p.539). In addition, in their recent review, Norsworthy et al. (2021) proposed that, unlike thriving, performance accomplishments are an *outcome* of flow, as opposed to a dimension of the concept itself (Brown et al. 2021a, 2021b).

Tedeschi and colleagues (2004, p.01) defined posttraumatic growth as ‘positive psychological changes experienced as a result of the struggle with trauma or highly challenging situations’. As posttraumatic growth is measure of change following adversity, it does not require a precursor of high levels of wellbeing nor performance. Although these authors do state that it is not the event itself per se but ‘the struggle with the negative event and the changes it has wrought’ (Tedeschi et al. 1998, 227). Thriving, on the other hand, is not necessarily the result of a struggle, nor is it predicated on adversity (Brown et al. 2017). It is possible, for instance, for an individual to thrive from a potentially traumatising event, without experiencing adversity. To exemplify, a first responder in a potentially traumatising event would be viewed as thriving if they are feeling competent, in control, and experiencing development and success. Posttraumatic growth would require the individual to experience the event as traumatising (as opposed to the feelings associated with thriving, above). Beyond these differences, the domains in which adaptation can occur differ between posttraumatic growth and thriving. Posttraumatic growth is centred on psychological adaptation in interpersonal relationships, new possibilities, personal strength, spiritual change and appreciation of life (Tedeschi and Calhoun 2004). Conversely, thriving encompasses physical and psychological wellbeing, performance, and success (Brown et al. 2017).

In a conceptual sense, psychological flourishing is perhaps most similar to thriving. Seligman (2011) proposed that, similar to how the concept of thriving is related to wellbeing, wellbeing plays a crucial role in contributing to flourishing. However, Seligman's (and others' subsequent) flourishing‐related work is focused predominantly on psychosocial and emotional wellbeing—as a result, that work does not also typically focus on an individual's *physical* state (Spreitzer et al. 2005). Brown et al. (2017) definition of thriving includes the components of emotional, psychological, and social wellbeing, and unlike flourishing, also focuses explicitly on an individual's physical state. Further, flourishing does not typically include consideration of one's ‘performance’ level in a given setting (Brown et al. 2017). Finally, Spreitzer et al. (2005) also distinguished between thriving and flourishing by suggesting that flourishing is a broader concept that does not necessarily emphasise learning. In contrast, it is argued that learning and development is an important component of the thriving construct.

Finally, it is worth mentioning wellbeing, which encompasses a high level of positive function in physical, emotional, and social domains. It is suggested that wellbeing also acts as a determinant of thriving, because high levels of wellbeing are necessary for an individual to experience the required development to ‘thrive’ (Brown et al. 2017). Notably, physical wellbeing is largely absent for other related concepts (e.g., flourishing, resilience).

In summary, whilst resilience and growth are in response to trauma or adversity, thriving represents an ongoing state of optimal functioning that may occur without the individual perceiving an event as traumatic. Further, thriving is not only distinct in that it includes a broader perspective on an individual's function, but it also includes a focus on physical wellbeing, and success (e.g. determined by perceived high performance) as an outcome. In particular, the framework proposed by Brown et al. (2017) may be a valuable concept as it suggests a more holistic approach to understanding and optimising human function that incorporates measures of physical wellbeing and performance, that underpin development and success.

### 1.3 Why Thriving Within High Stress and High Trauma Exposure Occupations?

A potentially traumatising event is defined in the Diagnostic and Statistical Manual of Mental Disorders (fifth edition; DSM‐5) as ‘exposure to actual or threatened death, serious injury, or sexual violence’ (American Psychiatric Association 2013, 271). Like most phenomena, traumatic incidents do not occur in isolation—individuals exposed to recurring trauma within their daily work are not immune to other forms of stress. These individuals often experience other ongoing stressors, such as lack of resources, high workload, or perceived low employee support (Lawn et al. 2020). Researchers have found both exposure to potential trauma and chronic stress increase the incidence of posttraumatic stress and alcohol use in emergency service personnel (see, e.g., Donnelly 2012). Therefore, it is important to pay attention to both potential trauma and the accompanying chronic stress present in these occupations. Following exposure to posttraumatic events, individual responses are varied and follow different trajectories depending on, for example, pre‐event risk factors, type of exposure, and individual social resources (see Alexander and Klein 2009; Pięta and Rzeszutek 2022).

As individuals in these occupations are regularly exposed to recurring trauma and stress and corresponding negative impacts, finding ways to facilitate thriving may have significant social, psychological, and organisational benefits. For example, thriving has been linked to overall positive health, as well as reductions in anxiety and depression (Spreitzer et al. 2005). Moreover, those considered ‘thriving’ in the workplace report better general health, fewer physical health issues, less psychological burnout, and less absenteeism (Spreitzer et al. 2012). Additionally, thriving employees express higher perceptions of competence, behave in a safer manner, and have a positive effect on their co‐workers (Jorritsma and Tolomei 2015). Evidently, it is important that efforts are made to facilitate thriving in individuals working in high stress and high trauma exposure environments, instead of merely helping individuals withstand trauma. Exploration of how individuals thrive in high stress and high trauma exposure roles (and potentially sustain a higher level of positive function over time) may provide significant benefits at an individual, organisational, and societal level.

In this scoping review, we aimed to explore whether, and how, thriving has been studied within populations working in high stress, high trauma exposure occupations. In turn, we aimed to identify potential gaps in the literature regarding the concept of thriving in diverse occupational environments in order to explore personal and environmental factors posited to contribute to thriving in people working in high stress, high trauma exposure occupations, and inform future intervention strategies.

## 2 Method

As the focus topic is a nascent area of research, a scoping review was deemed the most appropriate method to identify gaps, review and synthesise the research findings available (Arksey and O’Malley 2005). Further, a scoping review allowed us to clarify key definitions and concepts and identify factors relating to these concepts (Munn et al. 2018). For transparency and rigour, we adhered to the widely used methodological framework outlined by Arksey and O'Malley (2005) and the PRISMA extension for scoping reviews (Tricco et al. 2018). In line with Arksey and O'Malley's framework, this scoping review was conducted according to five broad stages: (1) identifying the research question; (2) identifying relevant studies; (3) selecting studies; (4) charting of data; and (5) collating, summarising, and reporting results. In this scoping review, we will investigate and report on how the concept of thriving has been defined, measured, and understood in the literature pertaining to high trauma and high stress exposure occupations.

### 2.1 Eligibility Criteria

Our review question and eligibility criteria were informed by the Population, Phenomenon of Interest, and Context (PICo) framework (see Stern et al. 2014). The phenomenon of interest was human thriving, and the population and context was individuals currently working within high stress, elevated threat and potentially traumatising work environments (See database search examples, Table 1).

**TABLE 1:** Database search examples.

PSYCH INFO	
#	Query
1	Psychological adjustment/or (workplace well being/or well being/or spiritual well being/or employee well being/) or thrive.mp. Or thriving.mp. Or flourishing.mp.
2	Limit 1 to (human and English language)
3	Emergency dispatchers/or emergency medicine/or emergency personnel/or crisis intervention services/or fire Fighters/or police personnel/or first responders/or rescue workers/or paramedics/
4	Limit 3 to (human and English language)
5	Exp × military personnel/or seafarer.mp.
6	Limit 5 to (human and English language)
7	Job stress/or traumatic stress/or secondary traumatic stress/or perceived stress/or work stress/or acute stress disorder/or chronic stress/or occupational stress/or psychological stress/or stress reactions/
8	Limit 7 to (human and English language)
9	4 or 6
10	2 and 8 and 9
**MEDLINE**	
1	Thrive.mp. Or exp × adaptation, psychological/or flourishing.mp.
2	Limit 1 to (English language and humans)
3	Exp × emergency responders/or emergency Responders.mp. Or (exp × military personnel/or military Personnel.mp.) or (exp × police/or Police.mp.) or emergency medical Dispatcher.mp. Or (exp × emergency medical services/or emergency medical Services.mp.) or seafarer.mp.
4	Limit 3 to (English language and humans)
5	Exp × psychological trauma/or psychological Trauma.mp. Or (exp × stress disorders, post‐traumatic/or stress disorders, Post‐Traumatic.mp.) or (exp × stress, psychological/or stress, Psychological.mp.)
6	Limit 5 to (English language and humans)
7	Exp × occupational stress/or occupational Stress.mp.
8	Limit 7 to (English language and humans)
9	6 or 8
10	2 and 4 and 9

The inclusion criteria for the articles include (a) qualitative, quantitative, and mixed method studies, (b) studies published in English in peer reviewed journals, and (c) participants were adults (aged 18 years or older) employed in high stress and high trauma exposure occupations. The inclusion criteria for these work environments were repeated exposure to elevated threat, and potential trauma as per the DSM‐5 definition—for example, professions such as first responders and military personnel (American Psychiatric Association 2013). Studies were included if the primary focus was on positive adaption in the target population. If the study had a dual focus (e.g. rates of posttraumatic stress disorder and posttraumatic growth in law enforcement), the study was included only if the research focus was positive adaption. The search criteria did not have a limitation on the year of study and the reference lists of the included articles were manually reviewed for further relevant articles.

Studies were excluded if the articles did not meet the above inclusion criteria, such as populations outside the target (e.g. children) or studies on thriving outside target occupational context (e.g. thriving in sport). Editorials, commentaries, books, reviews, conference abstracts, abstracts without full‐text available, and presentations were excluded. Grey literature sources (e.g. theses, white papers and conference papers) were excluded.

### 2.2 Search Strategy and Study Selection

**FIGURE 1:**
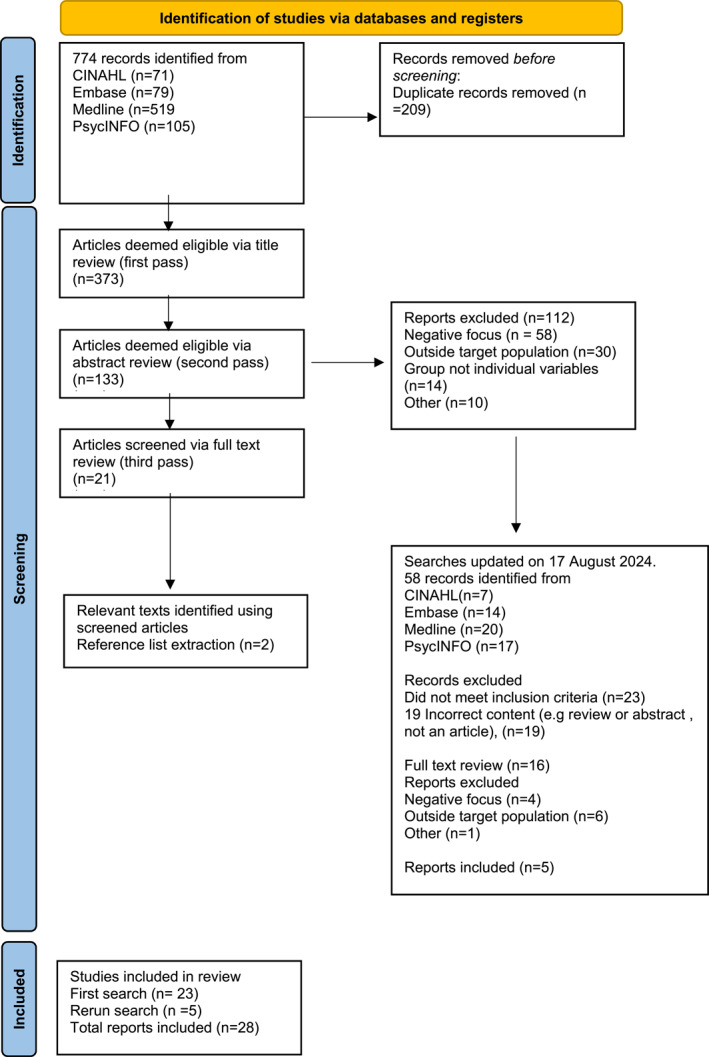
Study selection flowchart.

The search was conducted on 09 November 2022 and the search was rerun on 17 August 2024, with our selection process outlined in Figure 1. We conducted comprehensive searches of the Embase, CINAHL, Medline, and PsycINFO databases—in consultation with a university librarian to assist with our search strategy. Search terms were truncated, exploded, and modified for use on each database to suit the variations in design and configuration of databases—modifications to our search strategy were made without altering the principal content of the search. Two authors (SE and BF) conducted title and abstract screening to assess initial eligibility, and then all potentially relevant articles were examined in full‐text review to ascertain whether to include or exclude each article in the review. Articles were reviewed by the two authors independently, and a third author (KW) was consulted for conflict resolution at all stages of the review.

### 2.3 Data Extraction and Charting

Following study selection, data extraction was completed by the first author (SE). The following characteristics of each study were extracted: Year of publication, country, study design, participant age, number of participants, participant occupation, stress exposure information (type, time course and occupational stressors), factors of thriving (personal and environmental), concept under study (theory, process or model), positive function explored, measures of thriving, other measures in the study, time points, key findings, strengths, limitations, and definition of terms. An overview of the study characteristics and risk of bias assessment is outlined in Table 2. During the data extraction process, studies were reviewed and mapped against the components outlined as essential to meet the proposed defintion of thriving from Brown et al. (2017)—specifically, the joint experience of development and success and high levels of wellbeing and performance (Table 3). The Brown defintion was selected due to its inclusion of physical wellbeing and success (e.g., objective measure of performance), which clearly differentiates the framework from other related concepts and provides a potentially novel, and contextually flexible approach to understanding high levels of human functioning.

**TABLE 2:** Study details and risk of bias assessment results^a^.

Study details		Population				
First author + year	Country	N	Age (Years) [mean (range)]	Sex	Occupation/Setting	Risk of bias assessment
Adams et al. (2015)	Australia	*n* = 16	Mean = 41 Range = (24–57)	Male = 6 Female = 10	Emergency medical dispatchers	0.80
Arble and Arnetz (2017)	Sweden	*n* = 3656	67% in age range (30–55)	Male = Female = 751	First responders (coast guard, custom control, military, emergency medical services, fire and police)	0.86
Arble et al. (2018)	Sweden	*n* = 917 (56% response rate)	69% in the age range (30–55)	26% Female	Police officers	0.82
Austin et al. (2018)	United States of America	*n* = 54 (34% response rate)	Mean = 54	Not reported	61% paramedics 39% emergency technicians	0.59
Bauer and Herbig (2019)	Germany Austria Poland Czech republic	*n* = 72 24 western european 48 eastern european	Mean = 51.9 Range = (29–64)	Male = 72	Helicopter emergency medical service pilots	0.91
Chopko et al. (2018)	United States of America	*n* = 193	Mean = 41.6, Range = (23–63) Standard deviation = 9.2	93% Male	Patrol officer (65.3%), detective (19.7%), both patrol and detective (5.7%), administration (e.g., chief of police; 5.7%), and other (e.g., internal affairs; 3.7%)	0.73
Dåderman and Colli, 2014	Sweden	*n* = 101	Mean = 33 Standard deviation = 8	Female = 29 Male = 72	Police officers	0.95
Farero et al. (2024)	United States of America	1356 soldiers. 878 participated in at least two waves of data collection. 651 participated in the study in at least one wave, of these 651 soldiers, 596 participated in at least one post‐deployment data collection.	70.7% in the age range (18–30).	Male (95.2%)	National guard soldiers	1
Froutan et al. (2018)	Iran	*n* = 252	Mean = 28.9 ± standard deviation = 5.1 years Range = (21–50)	All male	Emergency medical services average work experience of 6.1 ± 4.8 years ranging from 1 to 25 years.	0.90
Harnett et al. (2021)	Australia	*n* = 506	10.7% in age range (20–30). 21.5% in age range (31–40). 37.9% in age range (51–60). 9.3% in age range (over 60)	Male = 258 Female = 248	Current or former police officers	0.95
Hellewell and Cernak (2018)	Canada	B01 *n* = 116 B02 *n* = 92 B03 *n* = 60	Cat 1: *M* = 36.93 Standard deviation = 1.8 Cat 2: *M* = 34.14 Standard deviation = 1.17 Cat 3: *M* = 34.0 Standard deviation = 2.35	B01 Male = 95 female = 21 B02 Male = 72 Female = 19 B03 Male = 47 Female = 13	Military personnel (pre‐deployment, during deployment to combat zone and post deployment)	0.86
Henson et al. (2022)	America and France	US (*n* = 409) France (*n* = 406), 815	American firefighters in age range (20–59) Mean = 37 Standard deviation = 8.4 French firefighters in age range (18–64) Mean = 37 Standard devaition = 10.7	2.7% of american participants and 13.2% of French participants were female firefighters	American firefighters were recruited in 28 different fire stations in charlotte, North Carolina. French firefighters were recruited in 14 fire stations in France in the departments of doubs and belfort.	
Hutchinson et al. (2022)	England	*n* = 160	Range = 20–64 Mean = 40.65 Standard deviation = 10.62	52.2% men 46.5% women 1.3% did not disclose	Ambulance officers—currently employed, frontline duties	0.68
Iwasaki et al. (2005)	Canada	*n* = 132	Mean = 39 Range = (24–61)	Male = 109 Female = 23	Police emergency response workers—urban centre	0.86
Jurišová (2016)	Slovakia	*n* = 62	Mean = 35.91 Range = (21–53)	Male = 30 Female = 32	Paramedics	0.95
Keech et al. (2020)	Australia	*n* = 134	Range = (25—59)	Male = 90, female = 42, undisclosed = 2	Police officers	0.91
Kirkcaldy et al. (1994)	United Kingdom	*n* = 533 (65% response rate)	Mean = 47.1 range = (28–58) Standard deviation = 4.3 years	Male = 522 (97.9%) Female = 11 (2.1%)	Police superintendents	0.68
Kwak and Im (2023)	Korea	*n* = 33 *n* = 16 (intervention) *n* = 17 (control group)	Mean = 33.56 Standard deviation = 5.21	Not specified	Firefighters	0.81
Larsson et al. (1988)	Sweden	*n* = 63 (initial) *n* = 54 (final)	Mean = 35.2	Male = 52 Female = 2	Police officers—Urban centre	0.82
Leppma et al. (2018)	United States of America	*n* = 113	Mean = 43.2 Standard deviation = 9.1	Male = 86 Female = 27	Police officers	0.91
Llorens et al. (2022)	Portugal	*n* = 1610	Mean = 35.2 Standard deviation = 9.1	98% male	Firefighters	1.0
Sandal et al. (1999)	Norway	Sample 1: *n* = 19 Sample 2: *n* = 25 Sample 3: *n* = 121 control	Sample 1: Mean = 27.11 Range = (21–38) Standard deviation = 4.97 Sample 2: Mean = 32.23 Range = (22–43) Standard deviation = 5.61 Sample 3: Mean = 20.30 Range = (19—27) *M* = 2905, standard Deviation = 4.30	Sample 1: Female = 1 Male = 18 Sample 2: Male = 25 Sample 3: Male = 121	Submariner	0.86
Sattler et al. (2014)	United States of America	*n* = 286	Mean = 38 Standard deviation = 11.	Male = 257 Female = 29	Firefighter/Emergency medical technician (64%), lieutenant/Captain (18%) Chief officer (9%) Firefighter/Paramedic (8%) Other (1%)	0.91
Senger et al. (2023)	United States of America	*n* = 155	Mean = 42.2 SD = 9.8	93.5% Male	Firefighters (career, volunteer, and combination departments)	0.95
Sudom et al. (2014)	Canada	*N* = 39 (initial) *N* = 34 (final)	Mean = 24 Range = (18–38) Follow up Mean = 31 Range = (24–47)	81.8% Male	Military personnel	0.77
Tomyn et al. (2015)	Australia	Mainstream sample *n* = 55,697	Mean = 49.92 Range = 18–76 Standard deviation = 17.29	51% Female		1.0
		Non child exploitation investigators *n* = 102	Mean = 40.27 Range = (21–55) Standard deviation 8.93	Male = 66		
		Child exploitation investigators *n* = 139	Mean = 38 Range = 24 to 58 Standard deviation = 7.49	62% Male 37% Female 1 Participant did not identify gender		
Veronese et al. (2017)	Palestine	*n* = 201	Mean = 34.3 Standard deviation = 8.46	42.8% male, 57.2% female	Aid workers/Professional helpers	0.86
Yoder et al. (2023)	United States of America	*n* = 119 quantitative *n* = 22 qualitative	Not specified	Female = 58 (48.7%) Male = 61 (51.3%)	Military enroute care nurses	0.95

^a^ As reported.

**TABLE 3:** Table of synonyms mapped to thriving definition and concepts.

Author/Year	Related concepts mapped to thriving (Brown et al. 2017)					Associated concepts utilised and definition
	Development	Success	Performance	Social wellbeing	Physical wellbeing or health	
Adams et al. (2015)	X					**Posttraumatic growth**—Positive changes in a person after experiencing trauma, where previous beliefs are challenged and cognitions of rumination and self‐disclosure are used to engage in growth processes (Cann et al.^2011^). Positive changes can occur in the domains of self‐perception, interpersonal relationships, and philosophies of life (Tedeschi and Calhoun 1996).
Arble and Arnetz (2017)	X				X	**Posttraumatic growth**—Growth as a process of emerging from a traumatic experience with a new perspective and attitude regarding one's life. (Tedeschi and Calhoun 1996) **Wellbeing**—Not defined. **Coping strategies**—Coping strategies involve two primary self‐regulatory techniques; approach or avoidance strategies (Fauerbach et al. 2009)
Arble et al. (2018)	X			X	X	**Posttraumatic growth**—A sense of revitalisation and new perspective on life following difficult events (Tedeschi and Calhoun 1996). **Coping strategies**—Coping strategies involve two primary self‐regulatory techniques; approach or avoidance strategies (Fauerbach et al. 2009).
Austin et al. (2018)	X					**Resilience**—Ability to recover from stressful situations. **Positive outlook**— Positive changes in outlook following adversity. **Posttraumatic growth**—Developing positive changes following traumatic or stressful events (Tedeschi and Calhoun 1996). **Wellbeing**—Not defined, however described as 'physiologically anchored”.
Bauer and Herbig (2019)			X	X		**Subjective wellbeing**—Not defined, however measured by world health organisation 5‐item measure. Work motivation—absorption, dedication, and vigour at work. **Work engagement**—The opposite of burnout, associated with increased self‐efficacy at work, better work performance, and better health and to depend on the availability of job resources.
Chopko et al. (2018)	X					**Posttraumatic growth**—Positive changes experienced in the aftermath of a traumatic event including aspects such as a greater appreciation for life, improved relationships, enhanced spirituality, an increased sense of personal strength and finding new paths in life (Tedeschi and Calhoun 1996).
Dåderman and Colli, 2014				X		**Sense of coherence**—A global orientation that expresses the extent to which one has a pervasive, enduring though dynamic feeling of confidence while living are structured, predictable and explicable; (b) the resources are available to one to meet the demands posed by these stimuli, and these demands are challenges, worthy of investment and engagement.
Farero et al. (2024)	X					**Perceived ability to handle stress**— Reflection of one's own ability to cope with stressful situations and perception of self. **Social support seeking**—Encompasses both instrumental support seeking (advice, help, or information) and emotional support seeking (sympathy, understanding, and moral support). **Purpose in life**—One's perception of having long‐term goals, direction, and meaning. **Social support**— Assessed as a contextual resource, indicating the amount of support available to the individual. **Optimism**— An enduring trait involving positive expectations for the future, distinct from posttraumatic growth constructs which involve transformational change. **Avoidant coping**—Marked by attempts to withdraw from, suppress, or deny experiences causing distress; generally considered maladaptive to posttraumatic growth. **Posttraumatic growth**—Positive psychological change experienced as a result of the struggle with highly challenging life circumstances.
Froutan et al. (2018)	X					**Resilience**—resilience is defined as embodiment of personal qualities that enable one to thrive in face of adversity. (Connor and Davidson 2003). Resilience as a constellation of characteristics can enable medical emergency personnel to adapt to their work‐related challenges (Jackson et al. 2007).
Harnett et al. (2021)					X	**Wellbeing**—A continuum ranging from ‘languishing’ to ‘flourishing’. well‐being is not simply the opposite of psychological distress (Keyes 2007; M. E. P. Seligman and Csikszentmihalyi 2000). **Positivity**—Consists of constructs of optimism, self‐compassion, gratitude and mindfulness.
Hellewell and Cernak (2018)	X		X	X	X	**Resilience**—The capacity to recover quickly, resist, and possibly even thrive in the face of direct and/or indirect traumatic events and adverse situations in garrison, training, and operational environments.
Henson et al. (2022)	X					**Core self‐evaluations**—Defined as a stable personality trait that encompasses people's fundamental evaluations about themselves, their abilities, and their personal control. **Posttraumatic growth**—Described as positive psychological change experienced as a result of struggling with highly challenging life circumstances.
Hutchinson et al. (2022)				X		**Resilience**—An individual internal resource that can be fostered by developing flexibility and adaptation to adversity (Froutan et al. 2018). **Satisfaction with life**—Subjective wellbeing and satisfaction with life. Those who are more satisfied have positive health outcomes, both physically and mentally (Diener and Chan 2011; Diener et al. 2018).
Iwasaki et al. (2005)				X	X	**Coping**—These researchers refer to lazarus and Folkman's work (Lazarus and Folkman (1985) define coping as; the cognitive and behavioural efforts made to manage specific external and/or internal demands that are appraised as taxing or exceeding the resources of the person. **Leisure**—What people do (leisure behaviour) and what people think and feel (leisure experience). Leisure is conceptualised as a buffer against the negative impact of stress to maintain physical and mental health.
Jurišová (2016)	X					**Posttraumatic growth**—Development is understood as a change, in which an individual advances to a higher level of his/her current adaption. A Significant positive in the cognitive and emotional life of an individual that may have external manifestations in one's behaviour.
Keech et al. (2020)			X	X	X	**Mindsets**—Refer to beliefs regarding the malleability of personal qualities and serve as a framework through which people make predictions about and judge the meaning of life events. **Stress belief model**—The belief that stress has positive implications for health and performance.
Kircaldy et al. (1994)					X	**Job satisfaction**— Not defined **Locus of control**—Not defined **Stress**—Not defined **Type a personality**– Not defined
Kwak and Im (2023)	X					**Posttraumatic growth**—Positive psychological changes subjectively perceived after traumatic events.
Larsson et al. (1988)			X			**Appraisal and coping**—Not defined, however, based on the work of Lazarus and Folkman (1985) implied by measures. Lazarus and Folkman (1985) define coping as; the cognitive and behavioural efforts made to manage specific external and/or internal demands that are appraised as taxing or exceeding the resources of the person.
Leppma et al. (2018)	X					**Posttraumatic growth**—Process in which positive and profound change occurs in one or more aspects of an individuals like following a traumatic event. Specifically, looked at posttraumatic growth development after stressful life events (both positive and negative).
Llorens et al. (2022)				X		**Work engagement**—Work (or employee) engagement is a positive, work‐related state of mind, that is characterised by vigour, dedication and absorption.
Sandal et al. (1999)					X	**Coping**—A cognitive process by an individual's expectations of being able to control the situation. **Positive outcome expectancy**—Defined as superior more effective coping, associated with reduced activation of stress response in central nervous system and accompanying responses in the motor, autonomic, endocrine and immune systems.
Sattler et al. (2014)	X					**Problem‐focused coping**—Involves taking direct action. **Emotion‐focused coping**—Involves managing stress by attempting to alter emotional responses to the situation (Folkman and Lazarus 1985). **Avoidance coping**—Involves avoiding or disengaging from the situation or processing the event. **Conservation of resources theory**—Individuals strive to acquire, maintain and protect valued resources and can experience stress when resources are threatened or lost. Resource caravans occur when resources are gained or lost across more than one domain. **Posttraumatic growth**—Critical incident exposure provides an opportunity for growth. Based on Calhoun and Tedeschi 2012).
Senger et al. (2023)	X			X	X	**Resilience**—Defined as the ability to not only survive but thrive in the face of adversity. **Hope**—Defined as a cognitive trait consisting of goals, pathway thinking, and agency. Goals are realistic, timely, and achievable; pathway thinking is the ability to think of multiple avenues to reach one's goal; agency is the motivation to reach one's goals using the identified pathways. **Posttraumatic growth**—Defined as positive psychological change experienced as a result of the struggle with highly challenging life circumstances. Posttraumatic growth is composed of five factors: Relating to others, new possibilities, personal strength, spiritual change, and appreciation of life. **Psychological wellbeing:** Conceived as a type of eudemonic well‐being, encompassing the deeper meaning of life and purposeful living and how that is connected to personal thriving. **Subjective wellbeing:** Conceptualised to include the more transient and affective parts of well‐being, composed of satisfaction with life, increased positive affect, and lower negative affect. **Social wellbeing:** Focuses on one's larger functioning in society and if the person finds connection and meaning in it.
Sudom et al. (2014)				X		**Resilience**—A personal characteristic or set of characteristics that protects individuals from the adverse effects of stress on wellbeing (e.g. Connor and Davidson 2003; Luthar et al. 2000).
Tomyn et al. (2015)		X		X		**Subjective wellbeing**—Wellbeing comprises people's affective and cognitive evaluations of their subjective life experience.
Veronese et al. (2017)	X			X		**Posttraumatic growth**—Positive psychological change after highly challenging life circumstances. **Subjective wellbeing**—This was operationalised three different indicators: Affect balance, psychological distress, general well‐being.
Yoder et al. (2023)	X					**Posttraumatic growth**—The experience of positive psychological change resulting from the struggle with major life crises and often results in changed priorities, appreciation for life, and spiritual and existential change. **Vicarious posttraumatic growth**—“positive changes that occur from vicarious or secondary traumatic exposure. **Resilience**—Marked by the ability to manage emotions and return back to the individual's baseline state, whereas PTG results in a transformed and/or improved sense of self.

### 2.4 Risk of Bias Assessment

Methodological quality of the studies was determined using a standard quality assessment tool for evaluating primary research articles developed by Kmet (2004). This tool uses a 10‐item checklist for qualitative studies and a 14‐item checklist for quantitative studies. Studies were scored based on the guidelines provided by Kmet (2004), with a total score calculated ranging from zero to one, with one indicated highest methodological quality (Kmet 2004). Consistent with the study selection procedure, studies were assessed for methodological quality independently by two authors (SE and BF). Discrepancies in quality assessment findings were resolved through discussion between the reviewers with a third reviewer (KW)—these discussions resolved all disagreements to create final methodological quality assessment scores (see Table 2). Studies were not excluded based on their risk of bias assessment.

## 3 Results

Seven hundred and seventy‐four records were initially identified following our database search, and two further studies were included from reference list searching. A total of 373 of these went through initial title screening, and 39 studies underwent full text review. Notably, no articles were found that utilised the specific term ‘thriving’ as the central concept of investigation in relevant occupational settings (i.e., high stress, recurring potential trauma). Our final sample retained 28 studies met the PICo criteria (Figure 1), and, despite not using the term thriving, focused on one or more components of thriving based on the definition from Brown et al. (2017) (see Table 3). The definition of thriving from Brown and colleagues was adopted due to the inclusion of physical wellbeing as underpinning development, and perceived and/or objective performance as a contributor to success, in turn differentiating this framework for other related. The 28 studies included in the review focused on positive adaptation within the target population—22 articles reported on a combination of personal (e.g., coping style) and contextual (e.g., social support) factors, and six articles were focused on personal factors exclusively.

The main reasons for exclusion were (a) contrary to the ‘success and development’ focus of thriving, the research focused on negative impacts and therefore did not meet our definition of thriving, (*n* = 62; e.g., the effect of trauma exposure in police and subsequent symptoms of depression), (b) the target population differed to our inclusion criteria (*n* = 36), or (c) the outcomes of the study were organisational variables (e.g., size of organisation) rather than individual variables (*n* = 14). An overview of the study characteristics is outlined in Table 2.

### 3.1 Stress and Trauma Exposure

Of the included studies, 20 utilised measures to assess participants' exposure to stress (e.g., Hutchinson et al. (2022) included the Perceived Stress Scale and the Impact of Event Scale). Five studies described the population's typical exposure to stress (e.g., participants' level of involvement in Hurricane Katrina; Leppma et al. 2018). Three studies did not specifically describe or measure the stress exposure of the targeted population. The majority of the studies (*n* = 25) focused solely on occupational stressors (i.e., stressors induced by being in that occupation, such as combat stress in soldiers; Hellewell and Cernak 2018). Additionally, three studies considered occupational stressors in conjunction with interpersonal stressors, such as family conflict or relationship issues (Adams et al. 2015; Iwasaki et al. 2005; Leppma et al. 2018). One of the above studies categorised the trauma and stressors as continuous due to the target population (Palestinian aid workers) living and working in constantly threatening conditions (Veronese et al. 2017).

The included studies consisted of 13 articles that focused on multiple types of stressors such as exposure to different traumas to self (e.g. Farero et al. 2024), or trauma to others (e.g. Yoder et al. 2023). Chopko et al. (2018) focused on the different impact of the type of stressor (i.e., potential threat to self or potential threat to others) in a population of police officers. Nine articles focused specifically on potentially traumatic events (e.g., combat stress in solders following an operation; Sudom et al. 2014). Tomyn et al. (2015) also studied vicarious trauma experience by police investigating child internet exploitation. Five studies did not specify the type of stressor exposure specifically, but did describe that their sample experienced high stress and exposure to trauma within their occupation. For example, the study conducted by Hutchinson et al. (2022) included participants described as front‐line ambulance officers; however, the authors did not specify the type of exposure the participants experienced.

### 3.2 Core Components of Thriving and Related Terms

All included studies explored one or more core component(s) of thriving, in line with Brown et al. (2017) definition (i.e., development, success, performance, or wellbeing). Table 3 provides a depiction of the related concepts included in final 28 studies, which also align with the core components of thriving.

### 3.3 Development

Sixteen articles focused on individual development following exposure to a stressor. The majority of these 16 articles (*n* = 13) aligned with Tedeschi and Calhoun's (2004) theory of posttraumatic growth—this concept closely maps to the *development* component of thriving. Ten of these articles included a form of the Post Traumatic Growth Inventory (PTGI) developed by Tedeschi and Calhoun (1996), a widely used measure with evidence of reliability and validity (Park and Sinnott 2018).

Arble and Arnetz (2017) and Arble et al. (2018) referenced posttraumatic growth theory for the basis of their studies; however, instead of using the PTGI to measure posttraumatic growth they constructed a self‐report inventory with a Cronbach's alpha of 0.75 or higher on the scales. Both Froutan et al. (2018) and Hellewell and Cernak (2018) studied development via resilience. Both studies specifically defined resilience as growth and the ability to thrive, thereby closely aligning with the framework of thriving as opposed to other definitions of resilience which refer to withstanding stress and pressure. The authors of both articles utilised the Connor‐Davidson Resilience Scale (CD‐RISC), one of the most widely used and validated measures of resilience across populations (Campbell‐Sills and Stein 2007Campbell‐Sills and Stein 2007). Three further studies explored resilience, but did not measure development as all three defined resilience as withstanding or bouncing back to normal function following adversity (Austin et al. 2018; Hutchinson et al. 2021; Sudom et al. 2014). Further, all three used the Brief Resilience Scale (BRS), a tool developed by Smith et al. (2008), which was designed to assess an individual's ability to adapt to stress and maintain wellbeing. For a review of the psychometric properties of the scales, see Ye et al. (2022). Senger et al. (2023) defined resilience utilising the same definition as Froutan et al. (2018) and Hellewell and Cernak (2018), and both measured resilience using the BRS. Farero et al. (2024) results suggest that growth and resilience occurred more frequently than psychological decline in their sample population. Further, Yoder et al. (2023) found posttraumatic growth was evident in 63% of the participants in their study.

### 3.4 Success and Performance

Only one study in the review included a measure of success via the Personal Wellbeing Index, which measures subjective ideals of success including a measure of ‘achieving in life’ (Tomyn et al. 2015). Four studies in the review referred to work performance as a variable or outcome (Bauer and Herbig 2019; Hellewell and Cernak 2018; Keech et al. 2020; Larsson et al. 1988).

The study conducted by Hellewell and Cernak (2018) was the only one that utilised objective measures of performance including saliva collection, spatial working memory, and reaction time. Keech et al. (2020) work focused on a model that posits that stress can enhance performance, with participants rating their belief in this notion and their subjective wellbeing. However, the subject's perception of their work performance or objective measures of performance were not reviewed. Bauer and Herbig (2019) measured work engagement and work conditions and stated that both factors are well known to affect work performance. They suggest that higher work engagement is associated with higher self‐efficacy at work, and better health and work performance. They included rating scales of occupational conditions and a measure of work engagement; however, work performance was not directly measured. Larsson et al. (1988) included a measure of quality of performance in a stressful encounter by Swedish police—this was a single, self‐report item of perception of performance within the Stressful Episode Questionnaire.

### 3.5 Wellbeing

Within the review, 11 studies included a measure of ‘subjective wellbeing’ or ‘wellbeing’—all were focused on wellbeing as an outcome variable rather than a precursive component of thriving. Although the definitions and measures differed slightly, wellbeing was generally measured on a continuum upon which an individual would rate their level of satisfaction in emotional, psychological, and social domains. This approach is in line with Brown et al. (2017) definition of wellbeing as a state of being or doing ‘well’ in life, categorised into physical, emotional, psychological and social domains. However, the physical component of wellbeing was largely not explored. From the limited work available included in this review, it is suggested that self‐reported levels of physical health are associated with perceived well‐being (Arble et al. 2018; Arble and Arnetz 2017). Both Harnett et al. (2021) and Iwasaki et al. (2005) used self‐report methods, reporting positive associations between physical activity and wellbeing. Two studies (Hellewell and Cernak 2018; Sandal et al. 1999) used biomarkers in their work. Hellewell and Cernak (2018) measured saliva biomarkers and neurocognitive performance in soldiers pre, during and post operational deployment. They concluded that some individuals thrive during stressful encounters, noting that this group within their sample showed an increase in cognitive performance and no change in the measured saliva biomarkers. In the other study to measure biomarkers of stress, Sandal et al. (1999) measured cortisol levels in submariners on deployment. Sandal et al. (1999) reported a rise in cortisol levels compared to a baseline group, indicating that submariners did find the mission stressful. They did reveal, however, that some of their participants demonstrated a lower cortisol response towards the end of the mission, suggesting a cortisol adaption that potentially relates to a more positive, adaptive personality style.

One study operationalised wellbeing differently—Llorens et al. (2022) specifically focused on wellbeing in the workplace. These authors defined wellbeing as a combination of negative and positive psychological states and used the Utrecht Work Engagement Scale and the Maslach Burnout Inventory. The remaining articles used commonly available measures with reliability and validity evidence, such as the Personal Wellbeing Index or the World Health Organisation Wellbeing Index. Two articles from Arble and measured wellbeing using a newly developed, domain‐specific self‐report measure derived from focus groups, with all items having a Cronbach's alpha of 0.75 or higher (Arble et al. 2018; Arble and Arnetz 2017).

### 3.6 Factors Contributing to Thriving

Six studies in the review outlined individual factors contributing to thriving (e.g., cognitive appraisal), and 22 studies focused on both individual factors as well as those within the individual's work environment (e.g., support from colleagues). We categorised the articles that described factors internal to the individual into those that explored (a) cognitive, (b) behavioural or (c) personality factors. The four studies investigating personality factors found that individuals with higher levels of agreeableness, optimism, and positive affect, and lower levels of neuroticism, had more adaptive outcomes (Farero et al. 2024; Froutan et al. 2018; Kircaldy et al. 1994; Sudom et al. 2014). Behavioural factors that were linked to better wellbeing included approach or proactive coping, being physically fit, or having a high level of physical activity (Harnett et al. 2021). Five studies indicated that, under some circumstances, avoidant coping may also be adaptive for example Arble and Arnetz (2017) found avoidant coping can assist wellbeing when used in conjunction with approach strategies in a study of police officers (Arble et al. 2018; Arble and Arnetz 2017; Harnett et al. 2021; Iwasaki et al. 2005; Keech et al. 2020). However, one study, conducted by Farero et al. (2024), reported avoidant coping predicted poor outcomes in their population of national guards. Seven articles considered the association of cognitive factors, such as appraisal style or cognitive flexibility, with thriving outcomes. e.g., two papers explored the relationship between deliberate rumination and posttraumatic growth. Kwak and Im (2023) reported no significant statistical difference in deliberate rumination and post traumatic growth in a sample of Korean firefighters, whereas Henson et al. (2022) reported a significant relationship between deliberate rumination and posttraumatic growth in American and French firefighters.

Ten articles positively linked posttraumatic growth (i.e., relating to others, new possibilities personal strengths spiritual change and appreciation of life) to higher wellbeing. Five studies documented that a challenge appraisal (or mindset) of stress leads to higher wellbeing (Dåderman and Colli 2014; Hellewell and Cernak 2018; Keech et al. 2020; Larsson et al. 1988; Sandal et al. 1999). Of the 22 studies that focused on contextual factors, all examined some form of social support—including social cohesion, peer support, or seeking social support. Social support was shown to be associated with positive adaption to stress or potentially traumatising events (e.g., see Farero et al. 2024). One study investigated frequency of work—either working part time, time between shifts, or vacations—reporting that positive outcomes (e.g. positive changes in outlook and post traumatic growth) were linked to more time away from work or time between shifts (Austin et al. 2018). Whereas Iwasaki et al. (2005) focused specifically on leisure and leisure pursuits as a predictor of health and found that frequency of leisure participation significantly predicted better immediate adaptational outcomes and greater mental and physical health in a sample of police officer and emergency response workers. Finally, two studies measured non‐psychological outcomes (Hellewell and Cernak 2018; Sandal et al. 1999). Both of these studies measured salivary cortisol as a stress marker, with Hellewell and Cernak (2018) also measuring neurocognitive performance, reaction time and attention. Broadly, they found that the participants that they deemed ‘stress thrivers’ had little change in hormone profile pre and post deployment and, improvement in cognitive function (Hellewell and Cernak 2018). In summary, our review revealed that the components of thriving as defined by Brown et al. (2017) of development and success and the underlying components of high performance and wellbeing have been studied within the target population. Further, cognitive, behavioural and personality factors have been suggested within the literature to contribute to individual thriving within this context. Contextual factors such as social support have also been suggested to contribute to positive, adaptive outcomes.

## 4 Discussion

The findings from our review revealed that human thriving as a central construct has not been explored in high stress and repeated trauma occupational settings. However, development, success, performance and wellbeing, all core components of thriving—have been captured in the research of related concepts such as resilience, posttraumatic growth, and coping (Brown et al. 2017). Drawing on insights from these related domains, our findings identified cognitive, behavioural, and personality‐related factors being important personal contributors, and social support as a critical environmental factor that may contribute to human thriving. This highlights a key gap the current understanding of thriving in the context of high stress and repeated trauma occupational settings but drawing on the findings from related concepts and of thriving in different contexts (e.g., sport), future research has the potential to advance our understanding of the value of the thriving framework in supporting human functioning.

Our synthesis reveals differences in the operationalisation of key concepts related to thriving within the literature. e.g., in some studies, resilience referred to the ability to recover from stressful situations (e.g., Austin et al. 2018), in others, resilience referred to a protective factor (e.g., Sudom et al. 2014), and in some work, resilience was posited to incorporate a level of functioning higher than before an adverse experience (which aligns with the notion of ‘development’ within Brown et al.’s, 2017, model of thriving; Froutan et al. 2018). Further explicating conceptual similarities between some researchers' operationalisation of resilience and thriving, Hellewell and Cernak (2018) and Senger et al. (2023) used the word ‘thrive’ in their definition of resilience, and measured resilience using the CD‐RISC (which defines resilience as *thriving in the face of adversity*; Connor and Davidson 2003). Other studies in our review measured resilience using the BRS (e.g. Austin et al. 2018), which instead focuses on one's ability to *maintain* function or bounce back to ‘normal’ following adversity. Aligning with Brown et al. (2021a, 2021b) work, resilience (which is posited to be conceptually different from thriving in that it requires exposure to adversity) may support the maintenance of thriving, rather than directly causing someone to thrive. However, it is evident that our understanding of resilience (and thriving) would benefit from consolidation of the way it is conceptualised in the literature.

Exploration of how people working in high trauma exposure occupations perceive potentially traumatic events is warranted—those who self‐elect into these occupations may perceive potentially traumatic events as a challenge to overcome, rather than a threat. It is posited that individuals who seek challenging experiences, and successfully overcome them, experienced an ‘advanced level of functioning’ and can be viewed as ‘thriving’ (Sarkar and Fletcher 2014). However, it may be the case that individuals who thrive in high trauma exposure occupations experience a stress inoculation effect (see Meichenbaum and Deffenbacher 1988) as a result of repeated exposure to traumatic events. These individuals, stemming from their prior exposure, may perceive new potentially traumatising events as controllable and less threatening, ultimately leading to feelings of competence, success, and further confidence in similar situations (Larsson et al. 1988). Additionally, Hellewell and Cernak (2018) highlighted that some people are ‘stress thrivers’, who demonstrate improvements in cognitive performance in high stress situations. Beyond how they perceive their environment, how people in high trauma exposure occupations cope after a stressful or threatening situation also holds importance in the context of thriving (Hellewell and Cernak 2018; Keech et al. 2020). e.g., although many researchers have demonstrated positive wellbeing and posttraumatic growth outcomes from approach coping (e.g., Arble et al. 2018; Jurišová 2016), others have also noted that avoidance coping and distraction play a role in how people in high trauma exposure occupations perform in situ during potentially traumatising events (e.g., Arble and Arnetz 2017). The interaction between high trauma exposure environments and thriving requires further explicit investigation—specifically, why (and how) some people thrive, and others develop posttraumatic exposure difficulties.

The above also has interesting implications on a theoretical level with the distinction between resilience and thriving and highlights a gap in the research literature. The definition resilience is a bouncing back to normal function, which then fails to capture those individuals who move beyond this and/or those who do not perceive the events as traumatic. In this way the concept of thriving is perhaps distinct and valuable to explore as it may capture both those who do and don't perceive a given experience or event and traumatic and thrive regardless. Exploration of how individuals thrive in high stress and high trauma exposure roles (and potentially sustain a higher level of positive function over time) may provide significant benefits at an individual, organisational, and societal level.

Of the articles reviewed 14 examined posttraumatic growth, either as the main focus or in conjunction with other concepts within the population. All of the studies used Calhoun and Tedeschi's (2012) definition and measures. In relation to Brown et al. (2017) definition of thriving, posttraumatic growth would be aligned to the development element of the definition. Although the studies found evidence of positive change in the populations, none of the studies measured whether or not the participants had a self‐perception or objective measure of success within their careers as would be a requirement to meet Brown et al. (2017) definition of thriving. The concept of thriving may be useful in exploring the relationship of perception of growth and how it manifests in a variety of real‐world domains (e.g. job performance or physical health). Interestingly, Calhoun and Tedeschi (2012) suggest that resilient individuals are less likely to experience posttraumatic growth as the traumatic event is less likely to impact their world view or shatter their assumptions and beliefs as suggested by Janoff‐Bulman (2010). This proposition has been reiterated by a number of authors such as Collier (2016). Within the articles reviewed, Bauer and Herbig (2019) found that on the whole, medical helicopter pilots' response profile was characterised by low to medium levels of perceived work stressors as well as high levels of motivation, wellbeing, and energy. Thus, we have a population of individuals who are reporting ‘doing well’ and potentially perceiving the exposure to high stressors at work as manageable. Exploring thriving within this high functioning population would be warranted.

In addition to the notion of ‘development’ explored above, ‘success’ is a proposed as a key facet of thriving (Brown et al. 2017), which is absent from the papers included in this review, potentially due to the lack of papers exploring thriving as a core component within this occupational context. Tomyn et al. (2015) asked child explotaition investigators about ‘achieving in life’, reporting that a higher sense of achieving in life predicted greater life satisfaction among those who worked in investigating child exploitation compared to those who did not. Of note, within this study, this particular domain accounted for twice the amount of variance of life satisfaction when comparing investigators who did and didn't work in child explotation. Beyond this example, there is a need for further investigation of ‘success’ within high trauma exposure occupations to provide insight into the applicability of thriving according to Brown et al. (2017) definition. Exploring ‘success’ as a measure of thriving is not without it challenges, e.g. is success considered an objective, externally referenced metric and/or related to the self‐perception and self‐evaluation of success? If using externally referenced metrics, such as those utilised within a sporting context by way of medals, wins or an objective time goal, there is also the concern around what would be considered valid in diverse settings and unpredictable environments. In high stress and trauma occupations such as emergency services, the measure of success may be highly influenced by organisational and/or environmental factors and therefore less accurate a representation of thriving at an individual level (e.g., in a firefighting context success as the number of fires effectively managed is contingent on the number and types of fires requiring management). In circumstances such as the above utilising measures which assess self or peer perception of success may be more relevant.

In exploring the applicability of Brown's framework with regards to ‘success’ in the target population it highlights the current gaps and opportunities for future research to further our understanding of supporting optimal human functioning in challenging occupations, both from the perspective of creating environmental facilitators and identifying and foster personal facilitators to thriving. e.g., high levels of wellbeing including quality of life factors such as access to healthcare, safety, clean air and subjective wellbeing underlie all of the adjacent concepts discussed in this review. An important feature of our synthesis was the inclusion of factors that contribute to positive adaptation and thriving. Personal factors influencing thriving included a sense of coherence or manageability when facing stress, optimism, acceptance, and gratitude (e.g. Larsson et al. 1988; Sudom et al. 2014). Many of these factors also appear to be linked with approach coping styles (e.g., Arble et al. 2018; Arble and Arnetz 2017), highlighting the importance of problem‐focussing coping for people working in high trauma exposure occupations. Physical health data (which aligns to the component of wellbeing) also appears to be a salient, yet under‐researched, contributor to thriving. Very few studies included in our review objectively measured physical health, despite strong existing evidence supporting the importance of physical health for wellbeing, positive functioning, and performance (e.g. Fedewa and Ahn 2011; Kramer 2020). Regarding environmental factors, social support or connection has been widely explored as contributing to thriving in this population, with findings from studies included in our review indicating that availability or usage of social support enabled more positive outcomes (e.g., Henson et al., 2022; Kwak and Im 2023). In particular, social support, connection, and shared humour among colleagues were reported to positively impact thriving‐related outcomes (e.g., Adams et al. 2015). Some researchers have posited that colleagues provide strong support networks for people in high trauma exposure occupations as they better understand the stressors involved with the job (Shakespeare‐Finch et al. 2015). Finally, having time or opportunities for rest and leisure between shifts appears to be an important environmental contributor to thriving, allowing people to use their non‐work time for coping mechanisms (Adams et al. 2015; Austin et al. 2018; Iwasaki et al. 2005).

The majority of studies included in this review relied on self‐report as the primary method for gathering data, with only two papers (Hellewell and Cernak 2018; Sandal et al. 1999) utilising any objective measures. This is a limitation of the review. In recent years, some researchers have discussed the possibility of self‐perceived psychological growth (e.g., often posttraumatic growth, but in this instance, thriving) actually representing positive illusions. If this is indeed the case, the strength of findings derived from self‐reports may be questioned. Infurna and Jayawickreme (2019) and Jayawickreme et al. (2022) have explored this issue within the research literature on post traumatic growth and subsequently question the strength of the findings. Researchers such as Infurna and Jayawickreme (2019) argue that this illusory growth may be due to emotional bias and/or the interplay of the individual and/or their specific cultural context (see also Jayawickreme et al. 2022; McLean and Syed 2016). We encourage further exploration of illusory biases, and more specifically, research methods and approaches that can be used to mitigate the risk of illusory biases occurring.

In response to the debate on positive illusion, authors such as Infurna and Jayawickreme (2019) have suggested future research encompass less reliance on self‐report and look to alterative methodology such as informant collaboration and longitudinal studies. This recommendation is also relevant to studies in this review, and is potentially easier to address when measuring thriving, as opposed to post traumatic growth, as it is a broader concept encompassing wellbeing, performance and success.

Although none of the articles reviewed directly measured thriving, all of the studies in this review measured or reviewed some aspect of positive function within the target population. This review highlights the need to explore the construct of thriving in high stress, high trauma exposure occupations with a particular focus on appropriate methodology to capture the nuanced and multidimensional nature of thriving. e.g., the concept of success ‘is typically evidenced through a variety of temporally and contextually relevant outcomes (e.g., attainment scores, improved cardiovascular capacity, wealth)’ all of which can be measured objectively (Brown et al. 2017, 168). Future research would benefit from undertaking methodology that can mitigate an illusory bias, utilising less self‐report and more objective measures as well as potentially utilising informant collaboration and longitudinal studies.

Our work was informed by Brown et al. ’s (2017) definition of thriving, which was developed within the context of sport. Although similarities exist between our population of interest and athletes (e.g., highly repetitive training procedures), they also differ (in that, the degree of threat, consequences of error and, personal risk of severe adverse events are much higher). The findings of our review suggest that both personal and environmental factors, including cognitive resilience, coping strategies, social support, and physical health, may significantly impact individuals' capacity to thrive under challenging conditions. The findings of this review indicate that it remains unclear whether the definition of ‘thriving’ suggested by Brown et al. (2017) is applicable to this population. The success aspect of the definition is largely absent in the research reviewed, and the definition of success itself within this context may look different. Differentiating between internal or individual perceptions of success from external measures may be a crucial consideration for thriving research. e.g., being able to distinguish between ‘doing well to the best my of ability’ which may not mean a positive outcome (e.g. a patient death). Further research is needed to determine whether, as Brown and colleagues claim, their definition of thriving is both flexible enough to cover across populations and specific enough to nuanced situations.

Most of the research reviewed studied the factors under consideration utilised quantitative methods. We recommend future research includes a qualitative approach to capture the complex, dynamic experiences of thriving among individuals in high trauma exposure occupations and utilising longitudinal research design would also assist in exploring the possible trajectories of thriving in the target population as well as the trajectory of any post traumatic depreciation. This has been studied in the posttraumatic growth research (e.g., see Pięta and Rzeszutek 2022). Finally, we recommend a greater focus on the physical component of thriving in future research.

## 5 Conclusions

Within the research on optimising human functioning in high stress, and recurring trauma occupation, thriving has not been investigated as the core concept to‐date. However, this review highlighted that previous research on related concepts within the specific occupational context, found individuals can experience a period of positive function in face of recurring potentially traumatising events. In turn pointing to the potential utility of thriving as a framework for understanding and optimising human function, on the premise that at is core the definition of thriving incorporates positive functioning in terms of development and success. Importantly, thriving is unique in its consideration of physical wellbeing and performance as underpinning the joint experience of development and success. Given the research to date has largely focused on a single dimension that forms part of thriving, such as psychological wellbeing, future research should explore the utility and adaptability of thriving as a complete concept. Given thriving is proposed to be a broader, more flexible model that encompasses both psychological and physical wellbeing and, includes positive changes without the precursor of negative events future research is warranted to explore and test this framework in various contexts. Additionally, the incorporation of physical wellbeing and success and performance measures as central to the definition also lends itself to methodological approaches not necessarily available to other concepts including objective measures of physical wellbeing, neurophysiological assessments of stress response, and objective metrics related to performance. Thriving as a construct is an important research focus as it embodies a holistic goal of high physical and psychological function including development, success, and wellbeing. Exploration of how individuals thrive in high stress and high trauma exposure roles (and potentially sustain a higher level of positive function over time) may provide significant benefits at an individual, organisational, and societal level.

## Conflicts of Interest

Prof Ben Jackson is a Deputy Editor at Stress and Health (Stress Management Interventions, Techniques and Processes). The additional authors declare no conflict of interest

## Data Availability

The authors have nothing to report.
